# Metabolic Functions of Peroxisome Proliferator-Activated Receptor *β*/*δ* in Skeletal Muscle

**DOI:** 10.1155/2007/86394

**Published:** 2007-01-03

**Authors:** Céline Gaudel, Paul A. Grimaldi

**Affiliations:** INSERM U636, Centre de Biochimie, UFR Sciences, Université de Nice Sophia Antipolis, Parc Valrose, 06108 Nice, France

## Abstract

Peroxisome proliferator-activated receptors (PPARs) are transcription factors that act as lipid sensors and adapt the metabolic rates of various tissues to the concentration of dietary lipids. PPARs are pharmacological targets for the treatment of metabolic disorders. PPAR*α* and PPAR*γ* are activated by hypolipidemic and insulin-sensitizer compounds, such as fibrates and thiazolidinediones. The roles of PPAR*β*/*δ* in metabolic regulations remained unclear until recently. Treatment of obese monkeys and rodents by specific PPAR*β*/*δ* agonists promoted normalization of metabolic parameters and reduction of adiposity. Recent evidences strongly suggested that some of these beneficial actions are related to activation of fatty acid catabolism in skeletal muscle and also that PPAR*β*/*δ* is involved in the adaptive responses of skeletal muscle to environmental changes, such as long-term fasting or physical exercise, by controlling the number of oxidative myofibers. These observations indicated that PPAR*β*/*δ* agonists might have therapeutic usefulness in metabolic syndrome by increasing fatty acid consumption in skeletal muscle and reducing obesity.

## 1. INTRODUCTION

The prevalence of adult obesity and obesity-associated metabolic disorders, including insulin resistance, glucose intolerance, hypertension, and dyslipidemia, has reached epidemic proportions in industrialized countries. The causes of the increase of this cluster of pathologies, known as the metabolic syndrome, are multiple and not totally elucidated. However, it is accepted that environmental factors, such as excess of food intake and lack of physical exercise, that characterize western lifestyle and lead to lipid homeostasis imbalance, are major contributors in the development of these pathologies. Lipid homeostasis requires a strict equilibrium between lipid availability and lipid consumption. In the normal situation, fatty acids coming either from food or from hepatic lipogenesis are utilized as energetic substrates in heart and skeletal muscles. Adipose tissue plays a central role in lipid homeostasis and can manage a transient increase in lipid availability by increasing the amount of stored triacylglycerol. However, long-term excess of dietary lipids and/or decrease of energy expenditure create a profound disturbance in this physiological equilibrium leading to a permanent increase in fatty acid availability and, on a long-term basis, to accumulation of triacylglycerol and other lipids in various tissues, such as adipose, liver, pancreas, and skeletal muscle. Such a lipid deposition leads to impairment of insulin responsiveness and metabolic dysfunction [1]. During the last decade, it has been demonstrated that adipocyte hypertrophy, a typical hallmark of adult obesity, results in a profound alteration of adipokine production and impairs the normal crosstalk between adipose tissue and the other organs increasing the metabolic disorders [2]. Several evidences clearly indicated that reducing lipid contents in blood and insulin-sensitive tissues is a crucial challenge to prevent metabolic syndrome. To reach this goal, lifestyle intervention has been shown to be an efficient strategy. For instance, weight loss, leading to a normalization of adipocyte size and adipokine secretion, and recurring physical exercise, promoting increment of energy expenditure in skeletal muscle and heart, have strong beneficial effects on insulin resistance and type 2 diabetes in human [3]. Because changing western lifestyle is very doubtful, pharmaceutical approaches mimicking the metabolic actions of weight loss and/or physical exercise should be of great interest. During the last 15 years, our knowledge of the molecular basis of lipid homeostasis regulation has been considerably improved and numerous studies have particularly demonstrated the roles of the peroxisome proliferator-activated receptors (PPARs) in the control of lipid metabolism, providing new ideas about the pharmacological treatment of metabolic syndrome.

## 2. PPARs: LIPID-ACTIVATED TRANSCRIPTION FACTORS AND REGULATORS OF LIPID METABOLISM

PPARs are ligand-activated transcription factors that belong to the nuclear receptor superfamily and play multiple physiological roles in several tissues. Three PPAR isotypes, *α* (NR1C1), *β*/*δ* (NR1C2), and *γ* (NR1C3), have been described so far. Each of the PPAR isotypes is encoded in a separate gene and exhibits tissue-selective expression patterns. PPAR*α* is mainly expressed in liver, heart, kidney, small intestine, and brown adipose tissue [4]. Several forms of PPAR*γ* have been identified with distinct expression patterns. PPAR*γ*2 is almost exclusively found in white and brown adipose tissues, while PPAR*γ*1 is expressed in several other tissues and cell types including intestine, placenta, and macrophages [5]. PPAR*β*/*δ* has a broad expression pattern in adult mammals, but it is abundantly expressed in small intestine, skeletal and cardiac muscles, brain, and adipose tissue [6, 7].

PPARs are organized in different domains. The amino-terminal domain is poorly conserved between the three isotypes and contains a ligand-independent transactivation function. The central domain, which is highly conserved, brings the capacity of DNA binding. The carboxyl-terminal region contains the ligand-binding domain and confers the ligand-dependent transactivation function. X-ray crystal structure analyses have revealed some important differences in the ligand-binding pocket of PPAR isotypes [8, 9]. These differences explain why PPAR isotypes can bind a large diversity of molecules and also display a relative selectivity for both natural and synthetic ligands.

PPARs heterodimerize with the retinoid X receptor (R-XR, NR2B) and bind to a specific DNA responsive element, called peroxisome proliferator response element (PPRE), found in a large number of genes encoding proteins involved in a variety of functions, including lipid and carbohydrate metabolisms, inflammation, cell proliferation, and differentiation [10, 11].

An important mark of PPAR transcriptional regulation is the interaction with cofactors. The unliganded PPAR/RXR heterodimer interacts with corepressors that exert transcriptional repression. It has been proposed that binding of the ligand promotes a conformational change that is permissive for interactions with coactivator proteins allowing nucleosome remodeling and activation of the transcription of target genes [8, 12]. Several corepressors and coactivators able to interact in a selective manner with the various PPAR isotypes have been described. Some of these cofactors are expressed in a tissue-specific manner and are controlled by physiological status in a given tissue. This selectivity of interaction could explain the differential tissue-specific transcriptional activities of the various PPARs and the activity level of a specific isotype depending upon the expression level of the cofactors in a given tissue or physiological situation.

It is now established that PPARs are lipid sensors and adapt the metabolic rates of various tissues to the concentration of dietary lipids. This role is related to the capacity of the various PPAR isotypes to bind fatty acids and fatty acid derivatives and to regulate the expression of several genes implicated in fatty acid uptake, handling, and metabolism in various tissues. Long-chain fatty acids, either saturated or unsaturated, appeared almost equally active for the three PPAR isotypes and, interestingly, the metabolism of the fatty acid is not required, as 2-bromopalmitate, a nonmetabolized fatty acid, appeared to be a potent PPAR agonist in preadipose cells [13].

Several fatty acid derivatives have been shown to be PPAR agonists. These molecules appeared to be more selective for the PPAR isotypes than fatty acids. For instance, the 15-deoxy-Δ12,14-PGJ2 (15d-PGJ2) is a selective PPAR*γ* agonist [14], leukotriene B4 and oleylethanolamide are activating selectively the *α* isotype [15, 16], and the prostacyclin is more active on PPAR*β*/*δ* than on the other isotypes [17]. However, as it is not possible to estimate the actual concentrations of fatty acids and fatty acid derivatives within the nuclear compartment, the physiological implication of these molecules as endogenous PPAR ligands remains an open question.

Due to their potential therapeutic interest for the treatment of metabolic disorders, several classes of PPAR synthetic ligands have been developed. Fibrates, used from several years as hypolididemic compounds, are specific ligands/activators of PPAR*α* [4]. Lipid lowering action of fibrates is mainly due to their capacity to upregulate, through PPAR*α* activation, several genes involved in hepatic fatty acid oxidation mimicking the effects of fasting that increases PPAR*α* expression in liver [18].

Thiazolidinediones [19] that are potent and specific activators of the *γ* isotype are used as insulin sensitizers. This action is paradoxically related to the adipogenic action of PPAR*γ*. It has been shown that thiazolidinediones promote a remodeling of adipose tissue by the recruitment of new and metabolically active adipocytes. These new adipocytes have beneficial effects by increasing the storage capacity of fatty acids and by normalizing adipokine secretion [20].

More recently, compounds able to specifically bind and activate PPAR*β*/*δ* have been developed and it has been shown that such compounds have beneficial metabolic effects in obese animals [21, 22]. The availability of these potent and specific agonists and the construction of appropriate cellular and animal models revealed the important roles of this PPAR isotype in lipid metabolism, especially in skeletal muscle, and pointed out the nuclear receptor as a potential target for the pharmacological treatment of metabolic syndrome.

Many studies revealed that PPAR*β*/*δ* agonists could be effective compounds to normalize several biological parameters perturbed during metabolic syndrome. Some of these studies were conducted by using the GW1516 compound that activates PPAR*β*/*δ* at very low concentrations both in vitro and in vivo with a 1000-fold selectivity over the other PPAR isotypes [23]. An interesting study by Oliver et al. has evidenced the beneficial actions of GW1516 administration in insulin-resistant obese monkeys [21]. Indeed, a 4-week treatment with the PPAR*β*/*δ* agonist increased high-density lipoprotein cholesterol, decreased low-density lipoprotein cholesterol, reduced the levels of small and dense low-density lipoproteins, and normalized insulin and triglyceride blood levels. Moreover, it was reported that the same molecule reduced adiposity and improved insulin responsiveness in diet-induced and genetically obese mice [22, 24].

The mechanisms involved in these beneficial actions of PPAR*β*/*δ* agonist administration to obese animals are not completely elucidated and, as the nuclear receptor is broadly expressed, it is likely that these actions are involving several tissues. However, during the last past years, several experimental evidences coming from both cell culture and in vivo studies have indicated that PPAR*β*/*δ* plays a central role in the regulation of lipid metabolism and adaptive development in skeletal muscle and that responses of this tissue could explain some of the antidiabetic and lipid-lowering actions of PPAR*β*/*δ* agonists in obese animals.

## 3. PPAR*β*/*δ*: REGULATORY ROLES IN MUSCLE METABOLISM AND PHYSIOLOGY

PPAR*β*/*δ* is several-fold more abundant than the other PPAR isotypes in rodent and human muscles [25]. Moreover, we have shown that long-term fasting [26] and endurance training [27], two physiological situations characterized by an increase in muscle fatty acid catabolism, increased PPAR*β*/*δ* mRNA and protein contents in mouse skeletal muscle. A similar PPAR*β*/*δ* upregulation was observed in human muscle after either long-term or short-term moderate exercise training [28–30].

Skeletal muscle accounts for about 40% of the body mass and, in this tissue, energy expenditure, insulin sensitivity, and fuel preference are highly affected by muscle work and myofiber composition [31, 32]. Depending upon their physiological roles, the different muscles contain variable percentages of specific myofibers that differ in both contractile and metabolic properties. Type 2b myofibers express fast isoforms of contractile proteins and synthesize ATP mainly from anaerobic glycolysis. Type 2a myofibers express fast contractile proteins, but contain more mitochondria, and are able to synthesize ATP from oxidation of glucose and fatty acids. Type 1 myofibers also have an oxidative metabolism and express the slow isoforms of contractile proteins. For instance, soleus muscle, which is implicated in endurance works, contains almost exclusively type 1 and type 2a oxidative myofibers, while the white gastrocnemius contains a majority of type 2b glycolytic myofibers and is implicated in short-term and intense exercise. Importantly, the myofiber composition of a given muscle is not fixed and is modified in some physiological or pathological situations. Endurance training promotes a fiber-type transition in human and rodents. In human muscle, moderate exercise induces a transition from type 2b to type 2a phenotype [33], while a more intense exercise is required for a transition toward type 1 phenotype [34]. Voluntary exercise increases type 2a myofiber percentage in several mouse muscles with or without hyperplasia, that is, increment in total myofiber number [35]. Sedentary life and type 2 diabetes lead to the opposite phenotype with a reduction of oxidative phenotype of various muscles [36, 37].

### 3.1. PPAR*β*/*δ* regulates fatty acid burning in skeletal muscle

Muoio et al. reported that exposure of differentiated human or rat L6 myotubes to a highly selective PPAR*β*/*δ* agonist or to a specific PPAR*α* agonist equally increased fatty acid oxidation and induced expression of several lipid regulatory genes, such as uncoupling protein 3 (UCP3), pyruvate dehydrogenase kinase 4 (PDK4), and carnitine palmitoyltransferase 1 (CPT1). These observations suggested a redundancy in the regulatory functions of both PPAR isotypes on fatty acid metabolism in cultured myotubes [38]. To directly establish the implication of PPAR*β*/*δ* in the control of lipid metabolism in muscle cells, we conducted gain-of-function and loss-of-function studies by overexpressing either native or dominant negative forms of the nuclear receptor in C2C12 myogenic cells. We showed that exposure of differentiated C2C12 myotubes to 2-bromopalmitate, a nonmetabolized fatty acid, or to GW0742, a specific PPAR*β*/*δ* agonist, upregulated expression of genes implicated in fatty acid uptake, handling, and metabolism, such as Fatty Acid Translocase (FAT/CD36), heart-Fatty Acid Binding Protein (h-FABP), and CPT1. Furthermore, the direct implication of PPAR*β*/*δ* in these regulations was established by the demonstration that the responses were, respectively, enhanced in PPAR*β*/*δ*-overexpressing cells and almost completely abolished in cells expressing the dominant negative form of PPAR*β*/*δ* [26]. A microarray expression profiling study confirmed these findings and showed that in L6 myotubes, activation of PPAR*β*/*δ* upregulated expression of a large panel of genes that control fatty acid transport, *β*-oxidation, mitochondrial respiration, and energy uncoupling [22]. Interestingly, Dressel et al. demonstrated that the various PPAR isotypes regulated different metabolic pathways in differentiated C2C12 cells. They reported that PPAR*β*/*δ* controlled fatty acid catabolism, while PPAR*α* was involved in the control of fructose uptake and glycogen metabolism, and PPAR*γ* controlled expression of genes implicated in glucose uptake and lipid synthesis [39].

Next to these data obtained with cultured myotubes, it was reported that administration of PPAR*β*/*δ* agonist upregulated expression of several genes implicated in lipid metabolism and fatty acid catabolism and reduced lipid content in mouse skeletal muscle [22].

The demonstration that PPAR*β*/*δ* agonists induced fatty acid burning in muscle, explains, at least partly, the beneficial effects of such treatment in obese animals, as it is well established that fatty acid catabolism is reduced in muscles from diabetic and obese animals and that lipid deposition is leading to insulin resistance, especially in muscle tissues [1, 36, 37]. Moreover, the generation of transgenic models for a muscle-specific overexpression of PPAR*β*/*δ* revealed another important and interesting function of the nuclear receptor in muscle physiology that could be very important for the understanding of the mechanisms implicated in the beneficial effects of PPAR*β*/*δ* activation.

### 3.2. Roles of PPAR*β*/*δ* in lipid metabolism and adaptive responses of skeletal muscle

To further investigate the roles of PPAR*β*/*δ* in muscle physiology, we have generated an animal model allowing a skeletal muscle-specific overexpression of the nuclear receptor [27]. In such an animal model, the PPAR*β*/*δ* protein content was increased by 4- to 6-fold early after birth in all types of myofibers, that is, oxidative and glycolytic, fast- and slow-twitch. Histological analysis revealed that the number of type 2a myofibers, that is, oxidative fast twitch, was increased in muscles from PPAR*β*/*δ*-overexpressing animals when compared to their control littermates. In tibialis anterior muscle and, to a lesser extent, in soleus muscle, this remodeling was due to an increase in total myofiber number, with a specific increase of type 2a myofibers, while in other muscles, such as plantaris and EDL, the increase in type 2a myofiber number was only due to conversion of type 2b to type 2a myofibers. These observations were confirmed by the demonstration that PPAR*β*/*δ* overexpression led to increased expression of genes implicated in fatty acid catabolism, such as citrate synthase, h-FABP, and UCP-2.

Another group investigated the effects of muscle-specific expression of a constitutively active PPAR*β*/*δ* (VP16-PP-AR*β*/*δ*) mutant form. Such animals displayed a more pronounced phenotype characterized by an increase of slow-twitch myofiber number in all types of muscles, including predominantly fast-twitch muscles [40]. The discrepancy between the two animal models could be due to the fact that the VP16-PPAR*β*/*δ* has a strongest transcriptional activity and upregulates expression of genes that are not affected by overexpression of the wild type PPAR*β*/*δ*. For instance, PGC-1, which plays a crucial role in conversion of fast-twitch to slow-twitch myofibers [41], is upregulated in muscles from VP16-PPAR*β*/*δ* mice [40] but is unchanged in muscles from PPAR*β*/*δ*-overexpressing animals [27]. However, it appeared that overexpression of either native or constitutively active PPAR*β*/*δ* forms has beneficial metabolic effects in mice by reducing adiposity, lowering lipid contents in several organs, and increasing insulin responsiveness [27, 40].

Collectively, these findings strongly suggested that overexpression and/or activation of PPAR*β*/*δ* mimics the actions of physical exercise on muscle remodeling and metabolism, at least in mouse. Several experimental evidences favor the hypothesis that PPAR*β*/*δ* plays a central role in adaptive response of skeletal muscle to endurance exercise. Daily moderate swimming exercise promoted PPAR*β*/*δ* upregulation in mouse skeletal muscle [27]. This increased expression requires several weeks of training, while it has been reported that in human muscle, a similar change in PPAR*β*/*δ* mRNA abundance takes place after shorter exercise period [28]. Moreover, VP16-PPAR*β*/*δ* mice display increased resistance to fatigue and running performance than their control littermates [40]. The molecular mechanisms that lead to the increased expression of PPAR*β*/*δ* in skeletal muscle during endurance training remain to be elucidated. Similarly, the molecular and cellular events that link the expression and activation levels of PPAR*β*/*δ* to myoblast proliferation and oxidative fiber typing remain to be characterized. However, it can be proposed that upregulation of the nuclear receptor is one of the first events leading to changes in the oxidative fiber number, while activation of PPAR*β*/*δ*, by natural or synthetic ligands, controls the degree of conversion of fast-twitch to slow-twitch phenotype.

## 4. CONCLUSIONS

During the few past years, the knowledge of physiological functions of PPAR*β*/*δ* has considerably increased and it is now established that specific agonists of the nuclear receptor may have therapeutic usefulness in metabolic syndrome. The actions of PPAR*β*/*δ* in skeletal muscle, that is, oxidative myofiber remodeling and increase of fatty acid burning capacity, may explain the beneficial effects of specific agonists on obesity and insulin resistance by limiting substrate availability for lipid synthesis and accumulation in adipose tissue and other insulin sensitive tissues. The muscle remodeling induced by PPAR*β*/*δ* activation may also affect the endocrine functions of skeletal muscle. It is now established that physical exercise is increasing fatty acid burning, but it is also changing the secretion level of muscle cytokines, called myokines, that control metabolic responses of other tissues, including adipose tissue [42]. Further studies are required to investigate the regulatory functions of PPAR*β*/*δ* activation on myokine production. Future work is also needed to clarify the roles of PPAR*β*/*δ* in other tissues that express the nuclear receptor at high levels, such as heart, intestine and brain, in order to prevent any side effects of PPAR*β*/*δ* activation.

Very importantly, level of experimental evidence is still restrained to animal models and a direct extrapolation of data obtained with rodent or primate models to the human context is risky as there are great differences in metabolic regulations between species. Clinical trials have been initiated and will provide important data regarding efficiency, tolerance, and safety in human for some PPAR*β*/*δ* agonists. The outcome of such clinical trials is eagerly awaited to confirm the regulatory roles of PPAR*β*/*δ* in human muscle physiology and metabolism.
